# Silicon’s Influence on Polyphenol and Flavonoid Profiles in Pea (*Pisum sativum* L.) under Cadmium Exposure in Hydroponics: A Study of Metabolomics, Extraction Efficacy, and Antimicrobial Properties of Extracts

**DOI:** 10.1021/acsomega.3c08327

**Published:** 2024-03-18

**Authors:** Justyna Walczak-Skierska, Aneta Krakowska-Sieprawska, Fernanda Monedeiro, Michał Złoch, Paweł Pomastowski, Mateusz Cichorek, Jacek Olszewski, Katarzyna Głowacka, Gaja Gużewska, Małgorzata Szultka-Młyńska

**Affiliations:** † Centre for Modern Interdisciplinary Technologies, Nicolaus Copernicus University, Wilenska 4, Torun 87-100, Poland; ‡ Department of Plant Physiology, Genetics and Biotechnology, University of Warmia and Mazury in Olsztyn, Oczapowskiego 1a, Olsztyn 10-719, Poland; § Experimental Education Unit, University of Warmia and Mazury in Olsztyn, Plac Łódzki 1, Olsztyn 10-721, Poland; ∥ Department of Environmental Chemistry and Bioanalytics, Faculty of Chemistry, Nicolaus Copernicus University, Gagarin 7, Torun 87-100, Poland

## Abstract

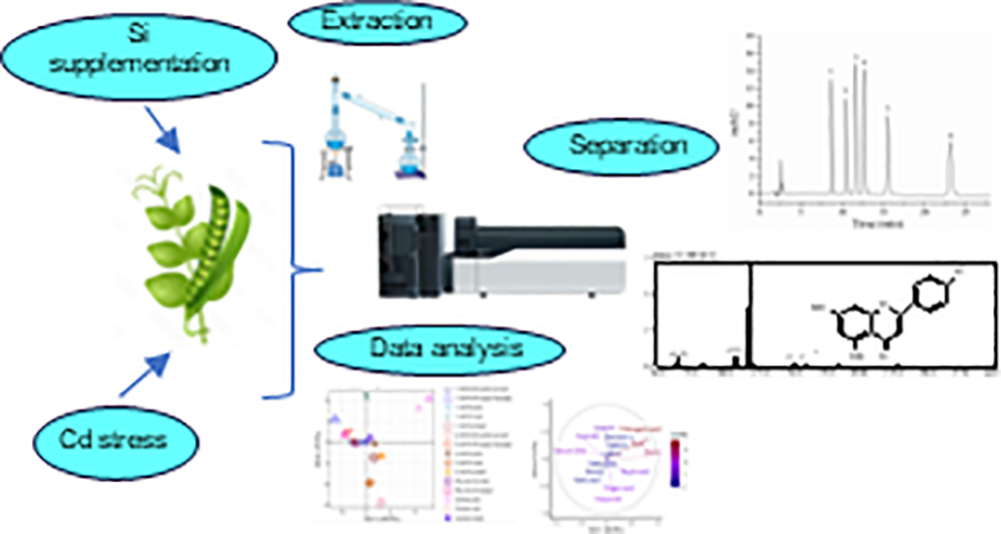

The current study aimed to investigate the impact of silicon (Si) supplementation in the form of Na_2_SiO_3_ on the metabolome of peas under normal conditions and following exposure to cadmium (Cd) stress. Si is known for its ability to enhance stress tolerance in various plant species, including the mitigation of heavy metal toxicity. Cd, a significant contaminant, poses risks to both human health and the environment. The study focused on analyzing the levels of bioactive compounds in different plant parts, including the shoot, root, and pod, to understand the influence of Si supplementation on their biosynthesis. Metabolomic analysis of pea samples was conducted using a targeted HPLC/MS approach, enabling the identification of 15 metabolites comprising 9 flavonoids and 6 phenolic acids. Among the detected compounds, flavonoids, such as flavon and quercetin, along with phenolic acids, including chlorogenic acid and salicylic acid, were found in significant quantities. The study compared Si supplementation at concentrations of 1 and 2 mM, as well as Cd stress conditions, to evaluate their effects on the metabolomic profile. Additionally, the study explored the extraction efficiency of three different methods: accelerated solvent extraction (ASE), supercritical fluid extraction (SFE), and maceration (MAC). The results revealed that SFE was the most efficient method for extracting polyphenolic compounds from the pea samples. Moreover, the study investigated the stability of polyphenolic compounds under different pH conditions, ranging from 4.0 to 6.0, providing insights into the influence of the pH on the extraction and stability of bioactive compounds.

## 1 Introduction

Legumes, like peas (*Pisum sativum*), are crucial crops, providing food and forage globally. Peas are highly esteemed and widely grown due to their unique flavor and nutritional richness. In 2021, global cultivation areas for green and dry peas reached 2.59 million hectares and 7.04 million hectares, respectively. Major producers include China (1.42 mln ha), India (0.56 mln ha), Pakistan (0.06 mln ha), USA (0.05 mln ha), and France (0.04 mln ha).^1^ Peas are renowned for their high protein content, reaching up to 31%, making them valuable dietary sources for both vegetarians and meat-eaters. They are particularly rich in lysine, an essential amino acid often deficient in cereals like wheat and rice.^2^ Moreover, pea protein is recognized for its low allergenic potential, making it an appealing choice for individuals with dietary sensitivities.^3^ Peas also offer a range of vitamins and minerals, including vitamins A, B_6_, C, and K, along with essential minerals like P, Mg, Cu, Fn, and Zn.^4^ Additionally, pea protein is noted for its low allergenic potential, making it suitable for individuals with dietary sensitivities.

The cultivation of pea crops faces significant challenges regarding the quality and safety of harvested produce, particularly concerning the accumulation of heavy metals and pesticides in agricultural soils. Cadmium (Cd) is a particularly concerning contaminant due to its threat to both human health and the environment.^5^ Cd infiltrates soil through various sources, including industrial activities like metal smelting and manufacturing, as well as the use of phosphate fertilizers, sewage sludge, and certain agricultural chemicals.^6−12^ Additionally, atmospheric deposition and improper waste disposal exacerbate cadmium pollution, especially in areas with historical industrial activities.^13−16^ This pollution poses risks to pea plants and other crops, impacting their growth, development, and physiological processes. Despite these risks, there is currently no comprehensive data on the minimum and maximum concentrations of cadmium in soil where peas or other legumes grow.^17,18^ Furthermore, the continuous use of Cd-contaminated soil has led to pollution in various dietary crops, including cereals, fruits, and vegetables, emphasizing the urgent need to address these sources to ensure the safety of crop cultivation for human consumption.^19−21^

Indirect evidence from global data indicates that legumes accumulate Cd within the range of 0.08–0.28 mg/kg fruit/seed dry weight, surpassing the maximum permissible values recommended by FAO/WHO for heavy metals in vegetables (<0.02 mg Cd/kg DW).^22,23^

Several studies, including those by Galal et al. (2021),^24^ Batool et al. (2022),^25^ and others,^26−29^ have illustrated the detrimental effects of Cd contamination on growth and yield parameters of pea cultivars, along with other pulses like pigeonpea,^30^ as well as globally significant crops such as wheat^31^ and tomatoes.^32^ Galal et al. (2021)^24^ investigated the accumulation potential of heavy metals in various organs of field pea and observed significant reductions in fresh and dry weights of pea plants, as well as pod production, in polluted (20.4 mg Cd/kg soil) solis compared to nonpolluted (0.2 mg Cd/kg soil) ones. Furthermore, Batool et al. (2022)^25^ found that Cd pollution significantly decreased traits related to growth, phenology, and biomass of pea plants, while also markedly increasing Cd contents in roots, shoots, and seeds compared to the control. Additionally, Cd contamination has been shown to highly affect the biomass and productivity of *Solanum lycopersicum* L. plants grown in soil irrigated with untreated industrial wastewater.^32^

Zhi et al.’s^33^ study on *Glycine max* L. revealed that low soil contamination with cadmium at 1.1 mg/kg did not affect plant growth and seed production. However, plants were capable of accumulating Cd beyond permissible levels, indicating potential hazards associated with the consumption of crops accumulating higher Cd concentrations in seeds or edible parts. In another analysis of metal concentrations in popular pulses grown near a Pb/Zn mine in Hunan, China, it was found that the long-term effects of spilled waste on the soil impacted human exposure through food chains. Variations between sites affected by multi-metallic stress resulted in differing Cd accumulations in seeds/fruits of various pulses, including adzuki bean 0.23–0.67 mg Cd/kg DW, string bean 0.34–0.67 mg Cd/kg DW, soybean 0.24 mg Cd/kg DW, mung bean 0.04 mg Cd/kg DW, and peanut 0.36–0.55 mg Cd/kg DW.^34^

Moreover, it was observed that when Cd concentration in soil was below 0.3 mg/kg, the yields of crops using legume-crop coplanting generally increased compared to monoculture. However, the Cd levels in edible parts of all tested crops, such as maize, tomato, cabbage, and pakchoi, were higher when cocultivated with legumes than those when grown alone. This suggests that legumes possess the ability to increase bioaccumulation and available Cd content in neighboring crops.^35^

Pea plants exposed to high levels of Cd in soil often exhibit growth suppression and stunted development,^24,25,36,37^ with symptoms like chlorosis in leaves indicating disruptions in chlorophyll production and photosynthetic activity.^38^ Cd interferes with various metabolic processes, leading to nutrient deficiencies and imbalances, further compromising plant health and productivity.^24,26,36,37^ Additionally, Cd accumulation in pea crops can impact the concentration of bioactive compounds in harvested seeds, such as antioxidants and phytochemicals, thereby reducing the nutritional quality of the harvested peas. These findings underscore the importance of managing Cd contamination in agricultural practices to ensure both crop productivity and food safety.^39^

Supplementation with silicon (Si) holds promise for alleviating the adverse effects of cadmium stress on pea plants. Si, abundant in the earth’s crust, enhances stress tolerance in various plant species by reducing Cd uptake and translocation among different plant organs.^40,41^ Studies indicate that Si treatments effectively decrease Cd concentrations in grains and straw of crops like cotton, cabbage, wheat, peanuts, and rice.^42^ Moreover, Si plays a crucial role in regulating physiological and biochemical processes in plants, enhancing enzymatic antioxidant activity, nutrient balance, and water status, thus promoting improved overall plant growth and development under stress conditions.^43,44^ Additionally, Si supplementation enhances the accumulation of both macro and micronutrients in plants, potentially enhancing the nutritional quality of pea crops.^45^ These findings align with Lide & Frederikse’s observation that Si primarily exists in a nontoxic form within an environmental pH range of 4.0–8.5, with a minor proportion as soluble monomeric silicate ions.^46^ Furthermore, the significance of Si in plant nutrition extends beyond its isolated role, as it interacts synergistically with other essential nutrients and elements such as phosphorus, magnesium, nitrogen, and heavy metals, contributing to the optimization of plant growth and health.^41,47^

Silicon supplementation enhances nutrient uptake and utilization in pea plants, contributing to their overall health and robustness. Various forms of silicon, such as sodium silicate (Na_2_SiO_3_), silicic acid (H_2_SiO_4_), colloidal silica gel (SiO_2aq_), and organosilicon compounds, are utilized in plant supplementation research to improve silicon’s bioavailability.^48,49^ However, the specific effects of Si supplementation on pea plants, particularly regarding its influence on the content of bioactive compounds, remain understudied. Investigating the optimal concentrations of Si compounds for peas and their potential to mitigate Cd uptake are crucial for reducing health risks associated with pea consumption and developing sustainable agricultural practices. Understanding the interactions among Si supplementation, Cd stress, and the biosynthesis of bioactive compounds in peas is essential for informed agricultural management.

Our study aimed to investigate the influence of Si supplementation on the metabolome of’Pegaz’ peas (*Pisum sativum* L.) under normal conditions and cadmium (Cd) stress.’Pegaz’ is characterized by its high sugar content and is well-suited for research due to its early maturity, short growth cycle, and dwarf growth habit.^26,27,50^ Previous studies on Cd’s effects on’Pegaz’ provided a basis for assessing Si’s role in mitigating Cd toxicity.^28,51^ We analyzed the effects of Cd and/or Si on growth, biochemical parameters, and Cd mobility in’Pegaz’ stems and roots.^28,51^ Si supplementation stimulated pea plant growth and reduced the bioconcentration factor of Cd (BCF) in both shoots and roots, indicating its potential to alleviate Cd toxicity.^51^ Furthermore, Si supplementation decreased glutathione reductase and polyphenol oxidase activity, suggesting its role in maintaining the cellular redox balance and preventing Cd-induced cellular damage.

Our study aims to investigate how silicon (Si) supplementation, specifically using sodium silicate (Na_2_SiO_3_), affects the levels of bioactive compounds in different parts of the pea plant, under both normal and cadmium (Cd) stress conditions. We hypothesize that Si supplementation enhances the plant’s stress tolerance, leading to changes in the accumulation and distribution of bioactive compounds, which could mitigate Cd-induced stress. Additionally, we explore the bioaccumulation and transfer of Cd within the plant across varying pH levels, aiming to understand Si’s role in reducing Cd uptake and translocation. Furthermore, we assess the stability of polyphenolic compounds in the plant under different pH conditions by utilizing various extraction techniques. Overall, our study seeks to deepen the understanding of how Si supplementation influences the biochemical, microbial, and adaptive responses in pea plants, particularly in the presence of Cd stress and fluctuating pH environments.

## 2 Materials and Methods

### 2.1 Chemicals and Reagents

The standards of flavonoids and phenolic acids were obtained from Sigma-Aldrich (Steinheim, Germany). Acetonitrile, formic acid (all LC-MS grade), and ethanol were purchased from Sigma-Aldrich (Steinheim, Germany). Ultrapure water was obtained from a Milli-Q water system (Millipore, Bedford, MS, USA).

### 2.2 Plant Material

The 6-week-old plants of *P. sativum* L. “Pegaz” were used for metabolomic analysis. Plants were grown in hydroponics, in Hoagland’s solution as a source of nutrients,^51,52^ at 3 different pH levels (4.0, 5.0, and 6.0). The seeds of the pea (PNOS, Ożarów Mazowiecki, Poland) after surface sterilization were germinated for 2 days in the dark. Subsequently, 2-day old uniform seedlings were transplanted for 1 week to a glass jar, followed by the transfer of the uniform plants to containers (4 plants per container) with 800 mL of a half strength Hogland’s solution. One week before treatment, the plants were transferred to a full-strength Hoagland solution. The 3-week-old pea seedlings were supplemented with 1 mM or 2 mM Na_2_SiO_3_ and treated with 50 μM CdSO_4_ for 1 week.^53^ The concentration of Cd and Si was decided from the previous studies.^26,53^ Three weeks after the beginning of the supplementation/treatment, plants were harvested for analysis. Nutrient solutions were changed twice a week over the course of the entire experiment to maintain a balance of defined acidity. The target pH was adjusted by using 1 N NaOH or 1 N HCl. In each experimental variant, there were 30 containers, resulting in a total of 120 plants per variant. The roots, shoots, and pods of the control and stressed and supplemented plants were air-dried separately at room temperature. The dried plant material was ground in a mill to homogenize and increase the specific surface area of the sample and then stored at room temperature without access to light.

### 2.3 Extraction Procedure

#### 2.3.1 Maceration

The ground dried plant materials (1 g) were soaked in ethanol (20 mL) for 24 h at 50 °C in the dark. The extracts were centrifuged (4000× *g*) (Eppendorf TM Centrifuge 5810R, Hamburg, Germany) and stored at −20 °C until further analysis.

#### 2.3.2 Accelerated Solvent Extraction (ASE)

To extract different parts of the pea, a Dionex ASE 350 system (Thermo Scientific, Waltham, MA, USA) was used, equipped with an autosampler carousel and a collecting tray for the sequential extraction of 24 samples. A 10 mL stainless steel extraction cell containing 1 g of the test material and glass beads was used, and 96% ethanol was used as the solvent for extraction. The extraction was carried out at 50 °C, 10 MPa pressure, for a static time of 10 min, with 3 static cycles. The extracts were centrifuged (4000× *g*) (Eppendorf TM Centrifuge 5810R, Hamburg, Germany) and stored at −20 °C until further analysis.

#### 2.3.3 Supercritical Fluid Extraction (SFE)

The extraction of target compounds was performed using a supercritical fluid extractor from MV-10 ASFE Systems (Waters Corporation, Milford, MA, USA), with a 10 mL extraction cell. The SFE chamber was filled with ground, dried plant material (1 g) and glass beads, and preliminary experiments were conducted to determine the optimal conditions. Extraction was carried out using scCO_2_ as a solvent and 96% ethanol as a co-solvent, at 50 °C, 20 MPa pressure, with a 30 min static period and a 10 min dynamic mode (continuous flow). The flow rates were set at 4 mL/min for scCO_2_ and 1 mL/min for 96% ethanol. The extracts were centrifuged (4000× *g*) (Eppendorf TM Centrifuge 5810R, Hamburg, Germany) and stored at −20 °C until further analysis.

### 2.4 HPLC-ESI-MS/MS Analysis

The common pea extracts were subjected to analysis of their polyphenol compounds using a Shimadzu LC-MS 8050 chromatographic system (Kyoto, Japan), which consisted of a binary solvent delivery system (LC-30 AD), controller (CBM 20A), autosampler (SIL-30A), and column thermostat (CTO-20AC). Separation was carried out using a Kinetex F5 column (100 × 2.1 mm, 2.6 μm, Phenomenex, Torrance, CA, USA). The Lab Solution 5.8 software was utilized for instrument control, data acquisition, and processing. The elution gradient was initiated with a mixture of 80% acetonitrile and 20% 0.1% formic acid in water (0.01–7.00 min, 0–80% ACN; 7–8 min, 80–80% ACN; 8–10 min, 80–0% ACN) at a flow rate of 0.4 mL/min and an injection volume of 8 μL. MS/MS analysis was conducted using positive and negative ionization mode on a Shimadzu triple quadrupole in the *m/z* range of 100 to 1000, equipped with an electrospray ionization (ESI) source. The ESI settings were configured as follows: nebulizing gas flow of 3 L/min, heating gas flow of 10 L/min, interface temperature of 300 °C, and DL temperature of 230 °C. All polyphenol compounds were monitored using the scheduled multiple reaction monitoring (MRM) mode (Table S1).

### 2.5 Preparation of Standard Solutions

To perform HPLC analysis, a stock solution of 15 standards, including phenolic acids and flavonoids, was prepared by dissolving each standard in pure methanol to a concentration of 100 μg/mL. Working solutions were then prepared by diluting the stock solution with methanol to obtain a concentration range of 0.00005–2.5 μg/mL, which were stored at −20 °C. Calibration curves were generated for each standard, and the limit of detection (LOD) and limit of quantification (LOQ) were determined for each standard by diluting the working solutions to a signal-to-noise ratio of three (S/N = 3) and ten (S/N = 10), respectively. Each experiment was repeated three times, and the mean ± standard deviation of the results was reported. An analysis of method validation is provided in Table S2.

### 2.6 Antimicrobial Property Assays

To evaluate the antimicrobial effect of the obtained MAC, SFE, and ASE extracts, disc diffusion as well as minimal inhibitory concentration (MIC) assay using a resazurin based *in vitro* toxicology assay kit was used as well as *Enterococcus faecalis* ATCC 51299 and *Escherichia coli* ATCC 25922 bacterial strains representing two main bacterial types, Gram-positive and Gram-negative, respectively. *E. faecalis* and *E. coli* belong to the fecal indicator bacteria (FIB) whose abundance in the soil is used for monitoring the risk of their transfer to the food chain resulting from the, among other things, manure application during agriculture processes. Thus, their control is vital for preventing foodborne illness. Before the experiment, ethanol (96%) was evaporated from the extracts. Then, the precipitate was dissolved in 10% DMSO – the volume depending on the mass of extract obtained – to get a concentration of 5 mg/mL. In order to prepare bacterial suspensions of 2 strains of pathogenic bacteria from 1-day cultures on MHA (Mueller Hinton Agar), an appropriate amount of biomass was taken by an ezy mesh into a saline solution to obtain optical density OD=0.5 McF (McFarland scale). From the suspension prepared this way, 100 to 9900 μL of saline solution was transferred and vortexed, and then 100 μL was applied to an MHA plate and spread. Plates prepared in this way were preincubated at 37 °C for 15 min to ensure complete absorption of the suspension by the agar. Discs [Ø 6 mm] were applied to the cultures prepared this way: 9 for each of the two plates prepared for a given bacterial strain. Then solutions of plant extracts (5 mg/mL) were applied to discs in a volume of 15 μL. Such prepared plates were incubated at 37 °C for 18 h, and then the presence or absence halo zones around the discs was measured. The MIC assay was performed based on the microdilution method using 96-well cell culture plates and Mueller–Hinton (MH) broth, according to the Clinical and Laboratory Standards Institute (CLSI) guidelines. The suspensions of 1-day bacterial cultures (0.5 McF diluted 100 times in saline solution) were mixed with the prepared concentrations of plant extracts in a ratio of 1:1 to obtain final concentrations: 2.5, 1.25, 0.625, and 0.313 mg/mL. Then, resazurin-based, 12 μL (final concentration of 45.8 μL/mL) of an *in vitro* toxicology assay kit was added to each well to determine bacterial viability. At the same time, a negative control was performed for the MH broth without the addition of a bacteria culture. The MIC value was determined based on the changed resazurin color from blue to pink. All measurements were prepared in triplicate. Bacterial cells without antimicrobial agents were used as a positive control.

### 2.7 Data Analysis

Data analysis was conducted in the R environment (R v.4.2.1), using an RStudio console (v. 2022.02.03, PBC, Boston, MA, USA). Heatmaps (”pheatmap” package) used as input the scaled average concentrations of bioactive compounds for each of the tested methods; for hierarchical clustering, association between samples was calculated based on the Euclidean distance. Principal component analysis (PCA) was performed using the package “factoextra” – in this case, the input data were the scaled concentrations calculated for all experimental replicates. Statistical comparison between control and supplemented plant material was performed using *t*-test (”stats” package). Normal distribution was previously checked by the Shapiro–Wilk test (”stats” package). Volcano plots were built by employing the “EnhancedVolcano” package.

## 3 Results and Discussion

### 3.1 Metabolomic Analysis of Biologically Active Compounds

The study focused on the metabolomic analysis of various morphological parts of peas, such as shoots, roots, and pods. The samples were subjected to different silicon supplementation conditions (1 and 2 mM Si), which is a known factor influencing plant metabolism. Additionally, the peas were exposed to a stress-inducing factor – cadmium (50 μM Cd, 1 mM Si and 50 μM Cd, and 2 mM and Si 50 μM Cd). Extracts derived from peas were obtained by using three different extraction methods: accelerated solvent extraction (ASE), supercritical fluid extraction (SFE), and maceration (MAC). Each of these extraction methods has its characteristic properties and can influence the composition and quantity of the obtained metabolites. Furthermore, extractions were performed for three pea crops. The pea crops were cultivated under different pH conditions (4.0, 5.0, and 6.0). pH conditions can influence the extraction efficiency and stability of certain metabolites. Therefore, conducting extractions under different pH conditions allows for a more comprehensive profiling of the pea’s metabolomic profile.

The application of a targeted HPLC/MS metabolite profiling approach enabled the identification of 15 metabolites, comprising 9 flavonoids and 6 phenolic acids. Comparing the applied analysis conditions, the highest number of bioactive compounds was identified at pH 5 and 6, while the lowest number was observed at pH 4 (Supporting Information Excel sheet). At pH 4, salicylic acid was primarily identified (for each extraction method), with a very minimal presence of biochanin A. The highest concentration of salicylic acid at pH 4 was observed in the root treated with 1 mM Si (0.6084 ± 0.0116 μg/mL for SFE, 0.3816 ± 0.0018 μg/mL for ASE, and 0.3260 ± 0.0063 μg/mL for MAC, respectively). The proper pH level is crucial, as it affects the availability of nutrients for growing plants. A pH level that is too high or alkaline can hinder nutrient absorption and lead to deficiencies. Hydroponic nutrient solutions typically start with a pH between 5.5 and 6.0, which is an optimal range for most crops.^54,55^

The analysis of the metabolomic composition of peas showed different contents of bioactive compounds in the analyzed morphological parts. The pea root exhibited the presence of 14 bioactive compounds, with two dominant groups: 9 flavonoids (flavon, hesperidin, quercetin, and other compounds were detected below the detection limit) and 5 phenolic acids (including chlorogenic acid, salicylic acid, caffeic acid, and minimal amounts of ferulic and sinapic acid). To gain a better understanding of the impact of silicon supplementation on the metabolomic profile of pea roots, we compared the roots of control plants with those supplemented with 1 and 2 mM silicon. Our research findings revealed that silicon supplementation had a significant influence on the content of specific bioactive compounds. For instance, we observed a decreased level of flavon, ferulic acid, quercetin, rutin, and salicylic acid depending on the applied pH and extraction method. Simultaneously, we noticed an increased content of chlorogenic acid, hesperidin, quercetin, and salicylic acid, depending on the pH and extraction method. We also examined the roots of plants subjected to cadmium stress and supplemented with 1 and 2 mM silicon. In the case of roots exposed to cadmium and supplemented with silicon, we observed a lower content of hesperidin and ferulic acid compared with plants treated with cadmium alone. However, we observed an inverse relationship for salicylic acid, quercetin, and chlorogenic acid, where the presence of cadmium resulted in increased levels of these compounds in pea roots (Supporting Information Excel sheet).

In the pea shoot, 14 bioactive compounds were identified, including flavon, chlorogenic acid, ferulic acid, synapic acid, biochanin A, quercetin, and salicylic acid. Additionally, apigenin, luteolin, rutin, esculetin, catechin, caffeic acid, and hesperidin were detected below the limit of detection. Increased concentrations of flavone, chlorogenic acid, ferulic acid, synapic acid, and quercetin were observed after silicon supplementation, at both pH 5 and 6. Silicon supplementation had an inhibitory effect on the content of salicylic acid. Furthermore, higher levels of salicylic acid, ferulic acid, and quercetin were found in the pea shoot of plants subjected to cadmium stress and supplemented with 1 and 2 mM silicon compared to plants treated with cadmium stress alone. Lower contents of biochanin A and hesperidin were observed in plants treated with cadmium stress and supplemented with silicon compared to those in plants treated with cadmium stress alone.

In the pea pod, flavon, chlorogenic acid, ferulic acid, quercetin, rutin, and salicylic acid were identified. Other compounds were present in very low quantities. These compounds may have potential health benefits and contribute to the anticancer and anti-inflammatory properties of pea pod. Supplementing with 1 mM silicon increased the content of salicylic acid, chlorogenic acid, and flavon (at pH 5) compared with control plants. However, at pH 6, the opposite trend was observed for the same compounds (Supporting Information Excel sheet).

Gallic acid was only identified in the root of plants supplemented with 1 mM silicon and extracted using the ASE extraction method. Caffeic acid was primarily detected at the limit of detection, both at pH 5 and 6, and for each extraction method. The highest concentration of synapic acid was observed in the roots of control plants (0.0094 ± 0.0001 μg/mL for SFE, pH = 6). Catechin was identified only in the roots of control plants (0.0097 ± 0.0001 μg/mL for MAC, pH 5) and in the pods of control plants (0.0082 ± 0.0001 μg/mL for SFE, pH = 6). Esculetin, luteolin, and apigenin were detected at the limit of detection, both at pH 5 and 6, and for each extraction method (Supporting Information Excel sheet).

Metabolomic studies have contributed to the identification of significant accumulation of metabolites that play a crucial role in the growth, maintenance, and development of plants under unfavorable abiotic stress conditions.^56^ Flavone, hesperidin, chlorogenic acid, ferulic acid, and quercetin are among the metabolites whose concentrations increase in response to Si. Their presence contributes to alleviating the negative impact of abiotic stressors on plants and maintaining their proper growth and metabolism.^57^ Additionally, the presence and quantity of identified biologically active compounds varied among different plant parts, such as shoot roots and pods, in both the silicon-supplemented group and the control group. These differences primarily stem from secondary metabolism processes, carbon accumulation during photosynthesis, phenylalanine metabolism, and amino acid metabolism. On the other hand, the observed metabolomic differences in the roots, both with and without Si supplementation, are a result of purine metabolism, amino acid metabolism, and the biosynthesis of secondary metabolites.^58^ In practical terms, this means that different plant parts respond differently to Si supplementation, affecting the types and quantities of biologically active compounds present in these parts. These compounds play a significant role in the plant’s functioning, its ability to adapt to changing environmental conditions, and its interactions with other organisms. Therefore, metabolomic studies allow us to better understand these differences and their effects on plant life.

The influence of heavy metals, such as cadmium (Cd), on various morphological parts of the pea plant is diverse.^59^ The aboveground parts perform autotrophic functions, synthesizing organic substances independently, while the underground parts have a heterotrophic character depending on externally supplied nutrients. The roots are responsible for the uptake of water and nutrients from the soil, while the shoot produce assimilates through the process of photosynthesis, in which plants convert carbon dioxide and light into carbohydrates.^60^ When it comes to the shoots, cadmium exerts an influence on diverse morphological, chemical, and physiological characteristics, including stomatal arrangement, efficiency of water utilization, moisture content, rate of evapotranspiration, levels of abscisic acid, stability of cellular membranes, and discrimination of carbon isotopes.^61,62^ The addition of Cd can induce changes in these traits, thereby influencing the functioning of shoots and photosynthetic processes. For example, cadmium can lead to stomatal closure to reduce water loss, ultimately resulting in a decrease in the rate of photosynthesis by reducing internal carbon dioxide concentration.^63^ Regarding the roots, the presence of Cd can lead to a reduction in root length and density, consequently limiting the plant’s ability to uptake water.^36^ Moreover, in the presence of this heavy metal, plants may respond by closing stomata to reduce water loss. These morphological changes directly impact the photosynthetic processes by reducing the internal carbon dioxide concentration, which is essential for photosynthesis. Studies on Cd accumulation have revealed that a significant portion of this element is absorbed by the roots and remains in root tissues.^64^ In this way, plants defend themselves against further Cd accumulation by restricting its translocation to other parts of the organism. This mechanism involves sequestering Cd through extracellular carbohydrates and cell wall deposition.^65^

Cd exposure in plants can lead to oxidative stress, resulting in the production of reactive oxygen species (ROS) and cellular damage.^66^ To mitigate this damage, reducing the oxidative stress in plants is crucial for maintaining plant health and productivity. One approach to achieve this is by applying Si, as demonstrated by various studies.^67−69^ Si can alleviate the adverse effects of stress, *i.e.*, on photosynthesis, by protecting the photosynthetic machinery and regulating photosynthesis-related genes^69^ or directly enhancing plant antioxidant defenses and reducing oxidative stress by limiting ROS production.^67^ Additionally, other methods, such as the use of phytohormones, beneficial bacteria like plant growth-promoting rhizobacteria (PGPR), or nanoparticles, have also been explored.^70−72^ Plant species and environmental conditions can determine the effectiveness of these approaches. Moreover, each of them has its own advantages and limitations. The phytohormones are involved in complex regulatory processes and interactions with other signaling molecules and, therefore, can produce variable results.^72^ Similarly, bacteria application may be difficult because it depends on the specific bacterial strains and plant species.^70^ Also, nanoparticles application can induce oxidative stress and cause toxicity.^73^ Among all of them, the application of Si offers a more practical and effective approach to reducing oxidative stress in plants.

### 3.2 Extraction Efficiency

**Figure 1:**
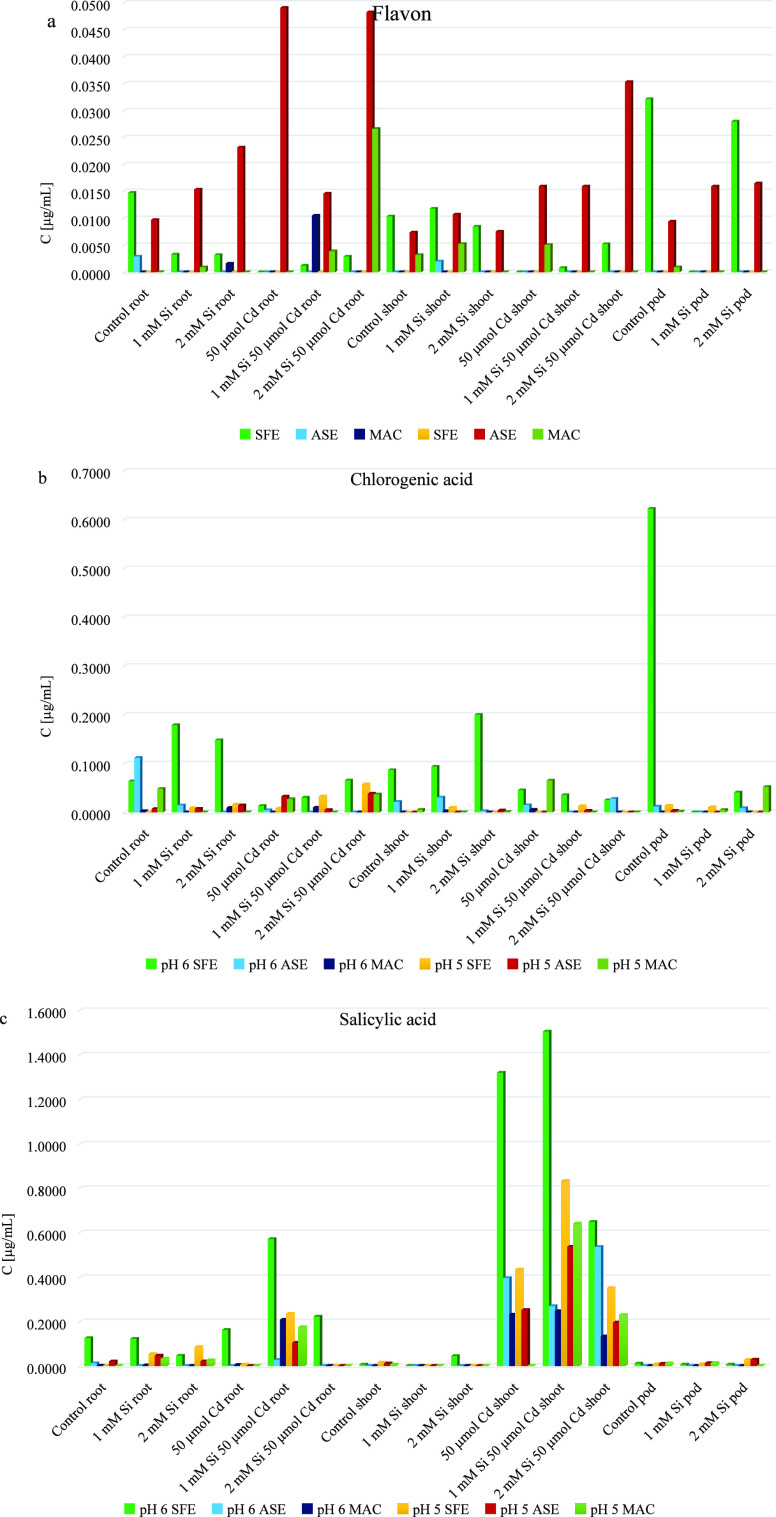
Efficiency of extraction for a) flavon, b) chlorogenic acid, and c) salicylic acid under various conditions.

The selection of a solvent and extraction conditions is a crucial step in the development of qualitative and quantitative analysis techniques for bioactive compounds in plant raw materials. The choice of extraction solvent plays a key role in predicting the qualitative and quantitative composition of isolated phenolic compounds.^74^ In order to compare the effectiveness of phenolic compound extraction from common peas, three extraction techniques were employed: the conventional method, accelerated solvent extraction, and supercritical fluid extraction. The conventional method involved macerating the morphological parts of peas in 96% methanol for 24 h, in darkness and at room temperature. The results of the experiment are presented in Figure 1, which depicts the content of three selected phenolic compounds (flavonoid, chlorogenic acid, and salicylic acid) in the shoot, root, and pod of peas at pH 5 and 6, using each of the three extraction methods. Upon analysis of the figure, it can be observed that the conventional method exhibited the lowest content of bioactive compounds at both pH 5 and 6. Specifically, for chlorogenic acid, maceration demonstrated the lowest efficiency compared to modern extraction methods.

The highest concentration of salicylic acid obtained through maceration was identified in the shoots of plants treated with Cd and supplemented with 1 mM Si, at pH 5. Similarly, the highest concentration of flavonoid obtained through maceration was observed in the root of plants treated with Cd and supplemented with 2 mM Si, at pH 5. These results suggest that for these two compounds, extraction using the conventional method is not the most effective.

In comparison to the conventional method, supercritical fluid extraction showed significantly better results for chlorogenic and salicylic acids, as evident in Figure 1. For these compounds, supercritical fluid extraction allowed for higher concentrations compared to the maceration.

Interesting results were also obtained by using accelerated solvent extraction. Analyzing Figure 1, it can be noticed that for flavonoid, the highest concentrations of this compound were observed when accelerated solvent extraction for plants that were cultivated at pH 5, for the shoot, root, and pod of plants treated with Cd and supplemented with Si.

The low efficiency of the maceration process results from various factors, such as the chemical composition of the extracted phenolic compounds, the number and position of hydroxyl groups, the size of the molecules, and interactions with other food components. This leads to a low selectivity and low recovery or extraction efficiency. Furthermore, the maceration process is time-consuming and requires significant labor. Additionally, the use of a large amount of organic solvents can be toxic and may remain in trace amounts in the extracts.^75^

### 3.3 Antimicrobial Properties

Evaluation of the plant extract antimicrobial properties is among the fundamental and most often analyzed factors that determine their benefits on the health of future consumers (controlling food-borne illness) and may indirectly indicate the content of plant secondary metabolites known for their antimicrobial properties, *e.g.*, tannins, terpenoids, alkaloids, and flavonoids.^76,77^

**Figure 2:**
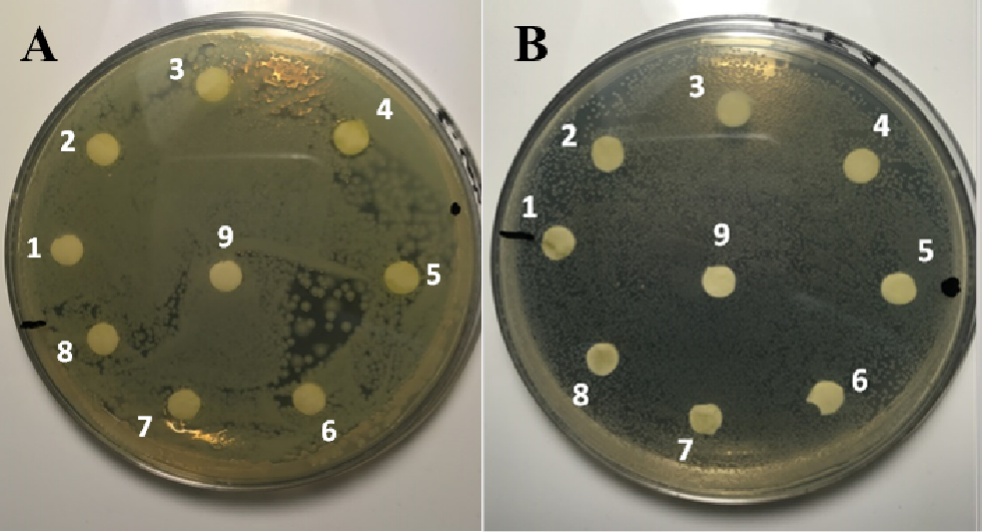
Exemplary plates obtained during antimicrobial effect evaluation using disc diffusion assay demonstrating no inhibitory effect of the common pea extracts on the growth of (A) *E. coli* and (B) *E. faecalis* cells. 1 – ASE leaves (control, pH 5); 2 – ASE leaves (2 mM Si, pH 5); 3 – ASE leaves (2 mM Si 50 μmol Cd, pH 5); 4 – SFE leaves (control, pH 5); 5 – SFE leaves (2 mM Si, pH 5); 6 – SFE leaves (2 mM Si 50 μmol Cd, pH 5); 7 – MAC leaves (control, pH 5); 8 – MAC leaves (2 mM Si, pH 5); 9 – MAC leaves (2 mM Si 50 μmol Cd, pH 5).

Performed antibacterial assays did not reveal any inhibitory effect of the common pea extracts regardless pH, plant organ, or extraction method used. Figure 2 presents an example of the plates obtained during the disc diffusion assay, which clearly shows no adverse effect of the extracts on the growth of both, Gram-positive *E. faecalis* and Gram-negative *E. coli* cells.

In the literature, many studies show the antibacterial activity of proteins extracted from the seeds of peas but much less about the antimicrobial properties of their extracts. Hadrich et al., during valorization of the extracts from the peel of pea, revealed their antimicrobial effect on the four different bacterial species: *Staphylococcus aureus*, *Escherichia coli*, *Pseudomonas aeruginosa*, and *Salmonella enterica* with a varied action degree.^78^ The inhibitory effect depended on the solvent type used during the maceration process; however, in most cases, it was lower compared to that of chloramphenicol and gentamycin use. It was clearly visible particularly for the MIC test when concentrations of the extracts needed to inhibit growth of the bacteria were tens of times higher (up to 100) compared to antibiotic solutions. Regarding the fact that for the disc diffusion assay tremendous concentrations of the extracts were used (100 mg/mL), the antibacterial effect of the extracts could be considered as low. Higher concentrations of the extracts used by the authors compared to our studies are the most probable explanation for the lack of inhibition observed in our work. Furthermore, the choice of the different plant organs also played a significant role in getting dissimilar observations.

### 3.4 Data Analysis

**Figure 3:**
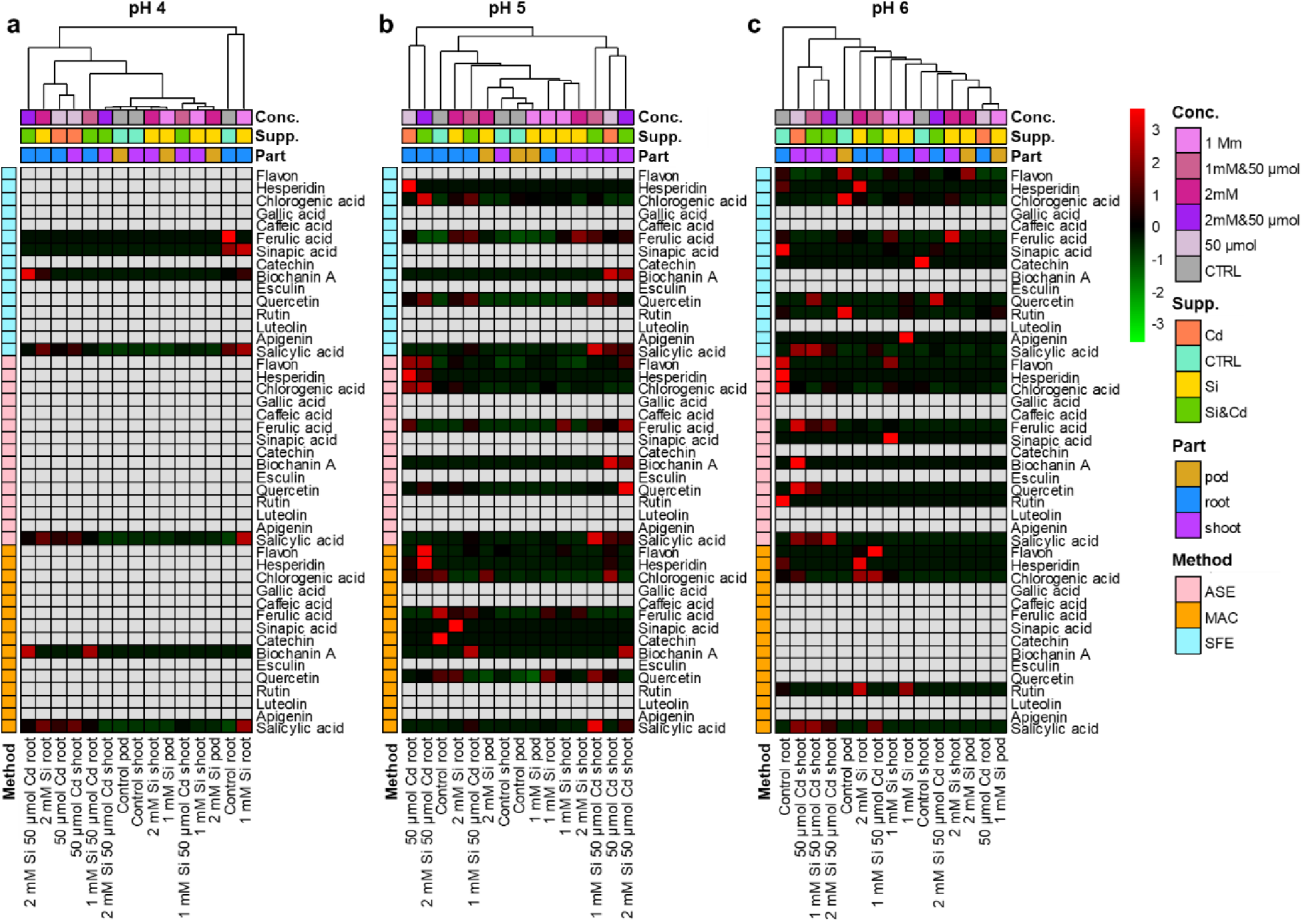
Heatmaps depicting average concentrations of bioactive compounds for different extraction methods performed at (a) pH = 4, (b) pH = 5 and (c) pH = 6. Empty cells refer to not detected compounds or those displaying responses < LOD.

Figure 3 shows heatmaps summarizing the efficiency of the tested methods. At pH 4 (Figure 3a), the lowest number of bioactive compounds was detected. At this pH, a slightly greater variability of analytes was measured when using the SFE method. Particularly, salicylic acid was the most stable compound at pH 4. At pH 5, MAC, followed by ASE, enabled the recovery of the greatest number of analytes (Figure 3b). Cluster analysis shows that at pH 5, it is possible to recover concordant amounts of bioactive compounds in root and shoot. Phenolic compounds in root were characterized by increased concentrations of flavon, hesperidin, and chlorogenic acid. In shoot samples, higher concentrations of biochanin A and salicylic acid. At pH 6 (Figure 3c), more analytes were recovered using SFE method, followed by ASE procedure. However, the levels of quantified compounds appear more distinct, according to the tested extraction method. In all cases, samples were not clustered according to the supplementation type or concentration, indicating that the effects of contamination on the observed content of bioactive compounds can be diverse and dependent on the method chosen to evaluate it.

**Figure 4:**
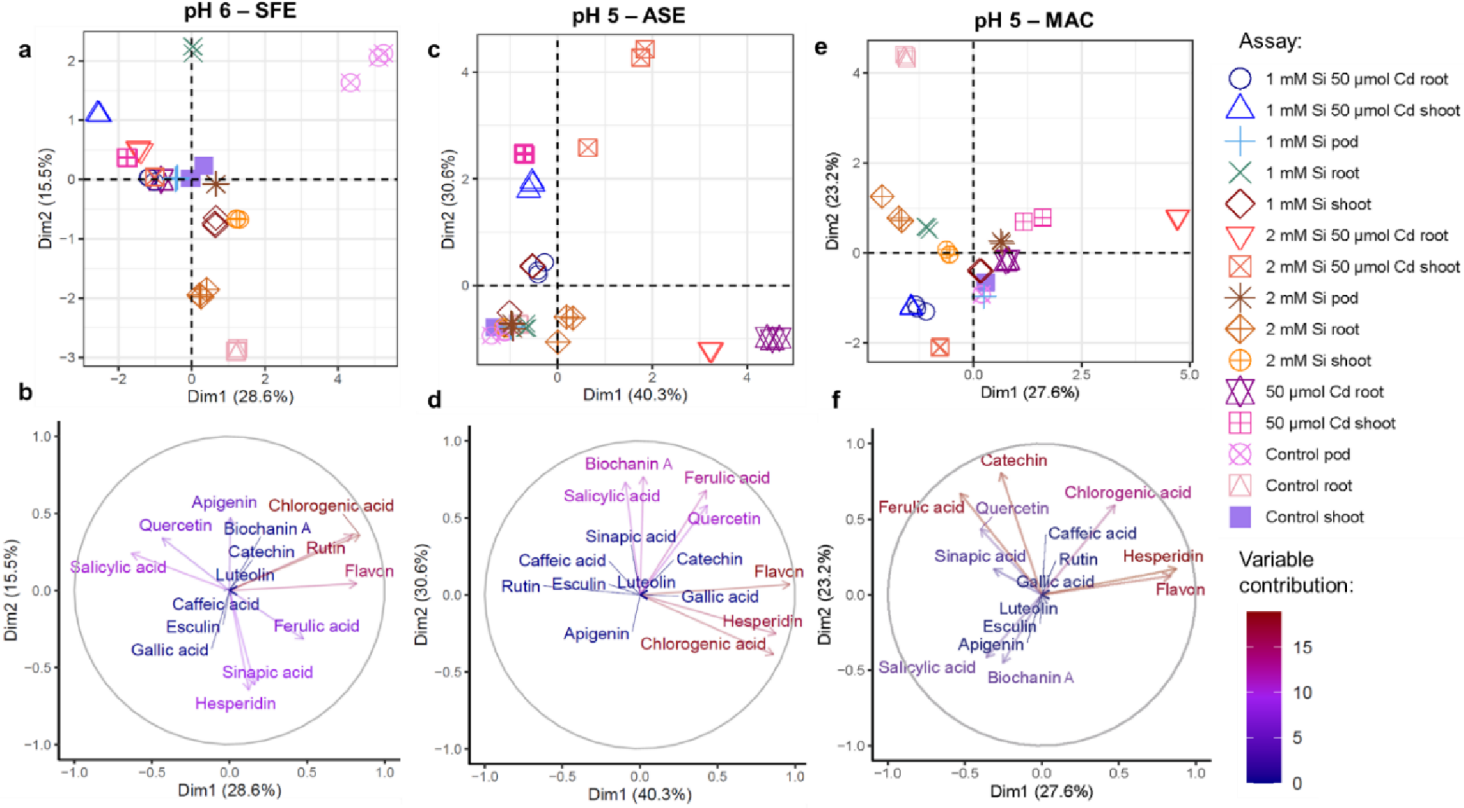
PCA of the measurements for bioactive compounds, obtained for the most efficient extraction methods (a, b) SFE at pH 6, (c, d) ASE at pH 5, and (e, f) MAC at pH 5. Upper panel: score plots; lower panel: variable plots.

PCA (Figure 4) was conducted on quantification data obtained under the conditions displaying the broader recovery of bioactive compounds. This method allow us to visualize patterns associated with the performed assays. Correlations between samples can be observed in the score plots (upper panel), while correspondence between compounds are plotted in the variables plot (lower panel). Finally, the overlaid coordinates of these plots allow us to visualize which compounds were enriched or depleted of which samples, according to the selected extraction method. As expected, replicates of experiments performed under the same conditions are mostly grouped together, indicating appropriate reproducibility. SFE at pH 6 (Figure 4a,b) provided notable recovery of chlorogenic acid and rutin in unsupplemented pods, while pods with 1 mM Si did not provide any enhanced level of bioactive compounds. In roots, supplementation with 1 mM Si decreased sinapic acid and hesperidin levels, while it increased apigenin content; Cd or the combined addition of Cd and Si led to increased levels of salicylic acid and quercetin. In shoots, 1 and 2 mM Si enhanced the recovery of ferrulic acid, while Cd addition increased salicylic acid and supplementation combining Si and Cd increased also quercetin. ASE at pH 5 (Figure 4c,d) allowed to assess more differences in relation to control root and shoot samples, which provided similar contents of phenolic compounds. In roots, Cd addition allowed the obtaining of increased levels of chlorogenic acid and hesperidin; similar findings were observed for supplementation with 2 mM Si + 50 μmol Cd. Shoots treated with Cd or Si in combination with Cd provided high recovery of biochanin A, salycilic acid, and ferrulic acid. MAC at pH 5 (Figure 4e,f) allowed to extract higher amounts of Sinapic acid and Quercetin after Si addition, for all plant parts. Addition of 1 mM Si + 50 μmol Cd increased levels of salicylic acid and biochanin A in roots and shoots; besides that, while 2 mM Si + 50 μmol Cd led to augmented levels of the same substances in shoot, in roots the levels of hesperedin and flavon were notably enhanced. On the other hand, Cd addition alone led to increased chlorogenic acid in roots and shoots.

**Figure 5:**
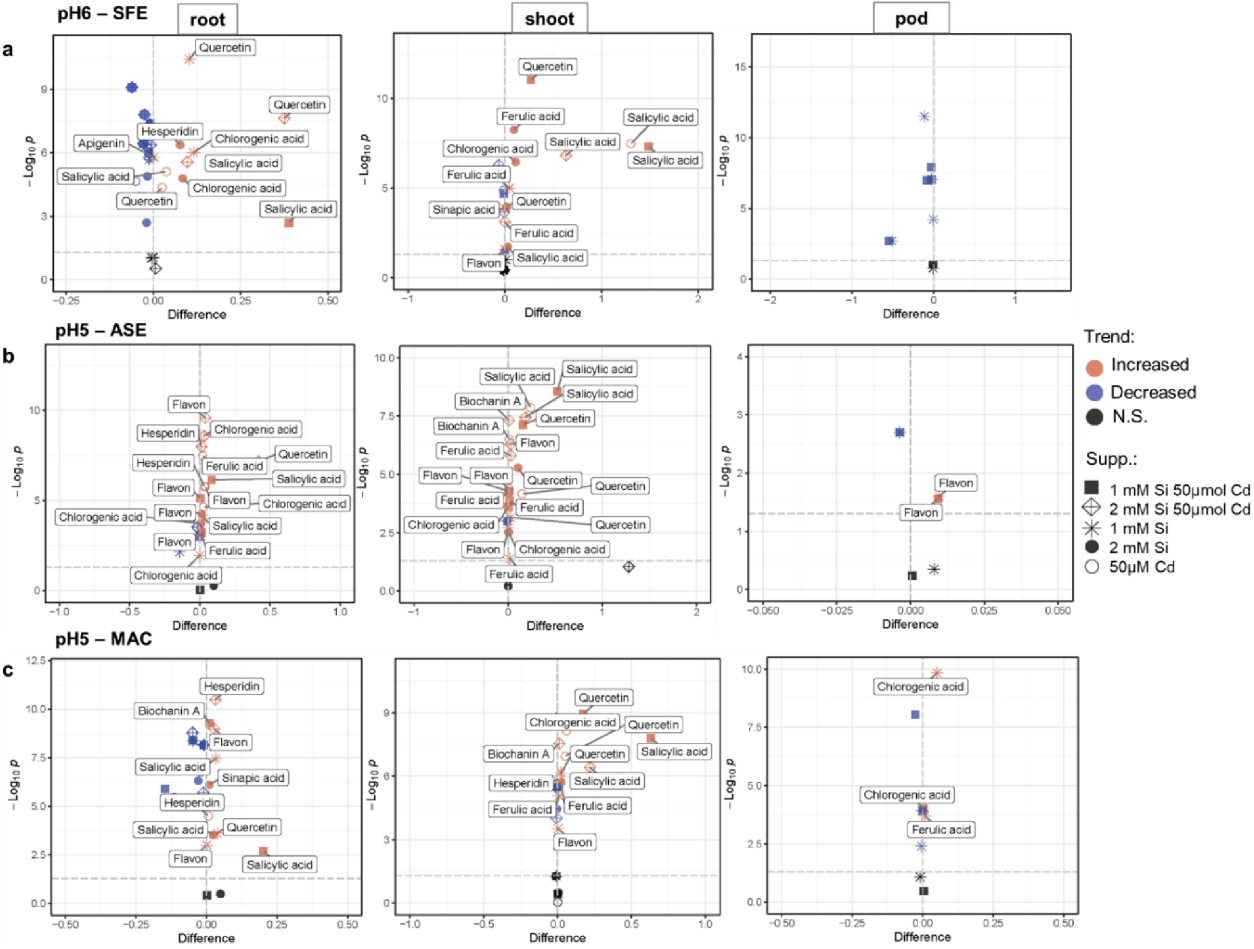
Volcano plots showing statistically significant changes in the concentrations of bioactive compounds extracted using (a) SFE at pH 6, (b) ASE at pH 5 and (c) MAC at pH 5, for root, shoot, and pod (left, middle, and right panels, respectively). Only compounds significantly increased in relation to controls are labeled. The dashed line indicates where *p* = 0.05.

Considering the usage of the proposed methods for the efficient extraction of bioactive products, an evaluation was performed of which compounds were significantly increased after the different supplementation tests. This evaluation is presented as volcano plots (Figure 5), in which the *x*-axis presents the difference in the concentrations in relation to controls and the *y*-axis displays the significance of these changes. In this way, Figure 5a–c show compounds enhanced by different types of supplementation, observed in accordance with SFE at pH 6, ASE at pH 5, and MAC at pH 5 (respectively). In general, it can be observed that Cd-induced stress reflected on greater levels of salicylic acid, quercetin, and hesperidin in relation to untreated plant material. Therefore, these compounds may have a function in the response to abiotic stress in peas. The combined addition of Cd and 1 mM Si increases at a greater fold change the concentrations of these same bioactive compounds. Considering that Si contributes for plant resistance against Cd, this supports the hypothesis that salicylic acid, quercetin, and hesperidin may have a role in plant adaptation to the environment. Besides that, plant materials treated with Cd and an even higher concentration of Si (2 mM) were enriched with other phenolic compounds, such as flavon (mainly in roots), as well as ferulic acid and biochanin A (mainly in shoots). The addition of 1 mM Si (solely) augmented the levels of chlorogenic acid, quercetin, salicylic acid, and flavon (in roots), and ferulic acid and flavon (in shoots). Si at 2 mM mainly led to the increase in the concentrations of chlorogenic acid and/or Ferulic acid in roots and shoots, as well as more diverse bioactive compounds such as apigenin (in roots) and sinapic acid (in roots and shoots). In pods, the material less rich in the surveyed bioactive substances, Si supplementation at 1 mM enhanced flavon recovery.

## 4 Conclusions

The conducted research demonstrates that silicon-mediated enhancement of bioactive compound production in peas offers a promising approach for augmenting the nutritional value and safety of leguminous crops cultivated in areas contaminated with heavy metals. Metabolomic analysis substantiates this claim, revealing that Si supplementation under cadmium (Cd) stress leads to increased biosynthesis and subsequent accumulation of flavonoids (such as flavon and quercetin) and phenolic acids (including chlorogenic and salicylic acid). Additionally, this study identified supercritical fluid extraction (SFE) as the most efficacious method for extracting polyphenolic compounds from pea samples. This finding is instrumental in guiding future research and the development of extraction protocols for polyphenolic compounds in peas and other plant species.

However, the formulation of targeted strategies to optimize Si supplementation for sustainable agricultural practices, particularly those aimed at minimizing heavy metal accumulation in crops, hinges on a deeper understanding of the mechanisms behind the Si-mediated enhancement of bioactive compound production in peas. Such an understanding is imperative for ensuring the safety and quality of food production systems involving peas and other leguminous crops and should be the cornerstone of future research endeavors.

## Supporting Information Available

All polyphenol compounds were monitored utilizing the scheduled multiple reaction monitoring (MRM) mode (Table S1); an analysis of method validation (Table S2) (PDF)
Concentrations of all identified compounds for three types of extractions are provided, as well as for each morphological part of the pea (XLSX)

## References

[ref1] FAO. The State of Food and Agriculture 2023 – Revealing the true cost of food to transform agrifood systems; FAO: Rome, 2023. DOI: 10.4060/cc7724en.

[ref2] Hara P.; Piekutowska M.; Niedbała G. Prediction of Protein Content in Pea (*Pisum sativum* L.) Seeds Using Artificial Neural Networks. Agriculture 2023, 13, 29 10.3390/agriculture13010029.

[ref3] Abi-Melhem R.; Hassoun Y. Is pea our hidden allergen? An American pediatric case series. J. Allergy Clin. Immunol. 2023, 2, 100090 10.1016/j.jacig.2023.100090. PMC10509857 37780801

[ref4] Ebabhi A.; Adebayo R.. Nutritional Values of Vegetables [Internet]. In Vegetable Crops - Health Benefits and Cultivation; IntechOpen, 2022. DOI: 10.5772/intechopen.101090.

[ref5] Alengebawy A.; Abdelkhalek S. T.; Qureshi S. R.; Wang M. Q. Heavy Metals and Pesticides Toxicity in Agricultural Soil and Plants: Ecological Risks and Human Health Implications. Toxics 2021, 9 (3), 42 10.3390/toxics9030042. 33668829 PMC7996329

[ref6] Wang J.; Wang L.; Wang Y.; Tsang D. C. W.; Yang X.; Beiyuan J.; Yin M.; Xiao T.; Jiang Y.; Lin W.; et al. Emerging risks of toxic metal(loid)s in soil-vegetables influenced by steel-making activities and isotopic source apportionment. Environ. Int. 2021, 146, 106207 10.1016/j.envint.2020.106207. 33197789

[ref7] Yin D.; Peng F.; He T.; Xu Y.; Wang Y. Ecological risks of heavy metals as influenced by water-level fluctuations in a polluted plateau wetland, southwest China. Sci. Total Environ. 2020, 742, 140319 10.1016/j.scitotenv.2020.140319. 32634688

[ref8] Zhang G.; Fan F.; Li X.; Qi J.; Chen Y. Superior adsorption of thallium(I) on titanium peroxide: Performance and mechanism. Chem. Eng. J. 2018, 331, 471–479. 10.1016/j.cej.2017.08.053.

[ref9] Chen R.; Chen H.; Song L.; Yao Z.; Meng F.; Teng Y. Characterization and source apportionment of heavy metals in the sediments of Lake Tai (China) and its surrounding soils. Sci. Total Environ. 2019, 694, 133819 10.1016/j.scitotenv.2019.133819. 31756835

[ref10] Chen Y.; Wang C.; Wang Z. Residues and source identification of persistent organic pollutants in farmland soils irrigated by effluents from biological treatment plants. Environ. Int. 2005, 31, 778–783. 10.1016/j.envint.2005.05.024. 16005065

[ref11] Maldonado V. M.; Arias H. O. R.; Quintana R.; Saucedo R. A.; Gutierrez M.; Ortega J. A.; Nevarez G. V. Heavy metal content in soils under different wastewater irrigation patterns in Chihuahua, Mexico. Int. J. Environ. Res. Public Health 2008, 5, 441–449. 10.3390/ijerph5050441. 19151441 PMC3700006

[ref12] Zhang Y. L.; Dai J. L.; Wang R. Q.; Zhang J. Effects of long-term sewage irrigation on agricultural soil microbial structural and functional characterizations in Shandong, China. Eur. J. Soil Biol. 2008, 44, 84–91. 10.1016/j.ejsobi.2007.10.003.

[ref13] Nabulo G.; Oryem-Origa H.; Diamond M. Assessment of lead, cadmium, and zinc contamination of roadside soils, surface films, and vegetables in Kampala City, Uganda. Environ. Res. 2006, 101, 42–52. 10.1016/j.envres.2005.12.016. 16527265

[ref14] Ogbonna P. C.; Okezie N. Heavy Metal Level and Macronutrient Contents of Roadside Soil and Vegetation in Umuahia, Nigeria. Terr. Aquat. Environ. Toxicol. 2011, 5, 35–39.

[ref15] Aoshima K. Itai-itai disease: Renal tubular osteomalacia induced by environmental exposure to cadmium—historical review and perspectives. J. Soil Sci. Plant Nutr. 2016, 62, 319–326. 10.1080/00380768.2016.1159116.

[ref16] Legrand M.; McConnell J. R.; Lestel L.; Preunkert S.; Arienzo M.; Chellman N. J.; Stohl A.; Eckhardt S. Cadmium Pollution From Zinc-Smelters up to Fourfold Higher Than Expected in Western Europe in the 1980s as Revealed by Alpine Ice. Geophys. Res. Lett. 2020, 47, e2020GL087537 10.1029/2020GL087537.

[ref17] Sigel A.; Sigel H.; Sigel R. K. O.. Cadmium: From Toxicity to Essentiality; Springer, 2013; pp 11. DOI: 10.1007/978-94-007-5179-8.

[ref18] Scott S. R.; Smith K. E.; Dahman C.; Gorski P. R.; Adams S. V.; Shafer M. M. Cd isotope fractionation during tobacco combustion produces isotopic variation outside the range measured in dietary sources. Sci. Total Environ. 2019, 688, 600–608. 10.1016/j.scitotenv.2019.06.269. 31254826

[ref19] Han Y.; Gu X. Enrichment, contamination, ecological and health risks of toxic metals in agricultural soils of an industrial city, northwestern China. J. Trace Elem. Miner. 2023, 3, 100043 10.1016/j.jtemin.2022.100043.

[ref20] Mazumder P.; Khwairakpam M.; Kalamdhad A. S. Assessment of multi-metal contaminant in agricultural soil amended with organic wastes, speciation and translocation – An approach towards sustainable crop production. Total Environ. Res. Themes 2023, 5, 100025 10.1016/j.totert.2023.100025.

[ref21] Romero-Estévez D.; Yánez-Jácome G. S.; Navarrete H. Non-essential metal contamination in Ecuadorian agricultural production: A critical review. J. Food Compos. Anal. 2023, 115, 104932 10.1016/j.jfca.2022.104932.

[ref22] Ali M. H.; Al-Qahtani K. M. Assessment of some heavy metals in vegetables, cereals and fruits in Saudi Arabian markets. Egypt. J. Aquat. Res. 2012, 38, 31–37. 10.1016/j.ejar.2012.08.002.

[ref23] Zhou H.; Yang W.-T.; Zhou X.; Liu L.; Gu J.-F.; Wang W.-L.; Zou J.-L.; Tian T.; Peng P.-Q.; Liao B.-H. Accumulation of Heavy Metals in Vegetable Species Planted in Contaminated Soils and the Health Risk Assessment. Int. J. Environ. Res. Public Health 2016, 13, 289 10.3390/ijerph13030289. 26959043 PMC4808952

[ref24] Galal T. M.; Hassan L. M.; Ahmed D. A.; Alamri S. A. M.; Alrumman S. A.; Eid E. M.; Ma Y. Heavy metals uptake by the global economic crop (*Pisum sativum* L.) grown in contaminated soils and its associated health risks. PloS One 2021, 16, e0252229 10.1371/journal.pone.0252229. 34086714 PMC8177654

[ref25] Batool T.; Javied S.; Ashraf K.; Sultan K.; Zaman Q. U.; Haider F. U. Alleviation of Cadmium Stress by Silicon Supplementation in Peas by the Modulation of Morpho-Physio-Biochemical Variables and Health Risk Assessment. Life 2022, 12, 1479 10.3390/life12101479. 36294913 PMC9605011

[ref26] Głowacka K.; Olszewski J.; Sowiński P.; Kalisz B.; Najdzion J. Developmental and Physiological Responses of *Pisum sativum* L. after Short- and Long-Time Cadmium Exposure. Agriculture 2022, 12, 637 10.3390/agriculture12050637.

[ref27] Orzoł A.; Gołębiowski A.; Szultka-Młyńska M.; Głowacka K.; Pomastowski P.; Buszewski B. ICP-MS Analysis of Cadmium Bioaccumulation and Its Effect on Pea Plants (*Pisum sativum* L.). Polym. J. Environ. Stud. 2022, 31, 4779–4787. 10.15244/pjoes/149259.

[ref28] Orzoł A.; Szultka-Młyńska M.; Głowacka K.; Krakowska-Sieprawska A.; Złoch M.; Buszewski B.. Changes in glutathione content and glutathione reductase activity in silicon supplementation during cadmium stress in pea (*Pisum sativum* L.). In Interdyscyplinarne badania w naukach przyrodniczych – innowacje, analizy i perspektywy (1), Kalbarczyk K; Danielewska A, Eds.; Wydawnictwo Naukowe TYGIEL Sp. z o. o: Lublin, Poland, 2023; pp 45 60. https://bc.wydawnictwo-tygiel.pl/publikacja/371E0025-0BD4-DBDA-B07B-9C795B0D0907.

[ref29] Saleem M. H.; Parveen A.; Perveen S.; Akhtar N.; Abasi F.; Ehsan M.; Ali H.; Okla M. K.; Saleh I. A.; Zomot N.; et al. Alleviation of cadmium toxicity in pea (*Pisum sativum* L.) through Zn–Lys supplementation and its effects on growth and antioxidant defense. Environ. Sci. Pollut. Res. Int. 2024, 31, 16111 10.1007/s11356-024-31874-5. 38198090

[ref30] Garg N.; Singla P.; Bhandari P. Metal uptake, oxidative metabolism, and mycorrhization in pigeonpeaand pea under arsenic and cadmium stress. Turk. J. Agric. For. 2015, 39, 234–250. 10.3906/tar-1406-121.

[ref31] Zhang G.; Fukami M.; Sekimoto H. Influence of cadmium on mineral concentrations and yield components in wheat genotypes differing in Cd tolerance at seedling stage. Field Crops Res. 2002, 77, 93–98. 10.1016/S0378-4290(02)00061-8.

[ref32] Ahmed D.-A. E.-A.; Slima D. F.; Al-Yasi H. M.; Hassan L. M.; Galal T. M. Risk assessment of trace metals in Solanum lycopersicum L. (tomato) grown under wastewater irrigation conditions. Environ. Sci. Pollut. Res. Int. 2023, 30, 42255–42266. 10.1007/s11356-023-25157-8. 36645601 PMC10067660

[ref33] Zhi Y.; He K.; Sun T.; Zhu Y.; Zhou Q. Assessment of potential soybean cadmium excluder cultivars at different concentrations of Cd in soils. J. Environ. Sci. 2015, 35, 108–114. 10.1016/j.jes.2015.01.031. 26354699

[ref34] Liu H.; Probst A.; Liao B. Metal contamination of soils and crops affected by the Chenzhou lead/zinc mine spill (Hunan, China). Sci. Total Environ. 2005, 339, 153–166. 10.1016/j.scitotenv.2004.07.030. 15740766

[ref35] Liu L.; Zhang Q.; Hu L.; Tang J.; Xu L.; Yang X.; Yong J. W. H.; Chen X.; Park S. Legumes can increase cadmium contamination in neighboring crops. PloS One 2012, 7, e42944 10.1371/journal.pone.0042944. 22905189 PMC3419222

[ref36] Haider F. U.; Liqun C.; Coulter J. A.; Cheema S. A.; Wu J.; Zhang R.; Wenjun M.; Farooq M. Cadmium toxicity in plants: Impacts and remediation strategies. Ecotoxicol. Environ. Saf. 2021, 211, 111887 10.1016/j.ecoenv.2020.111887. 33450535

[ref37] Almuwayhi M. A. Effect of cadmium on the molecular and morpho-physiological traits of *Pisum sativum* L. Biotechnol. Biotechnol. Equip. 2021, 35, 1374–1384. 10.1080/13102818.2021.1978318.

[ref38] Muradoglu F.; Gundogdu M.; Ercisli S.; Encu T.; Balta F.; Jaafar H. Z.; Zia-Ul-Haq M. Cadmium toxicity affects chlorophyll a and b content, antioxidant enzyme activities and mineral nutrient accumulation in strawberry. Biol. Res. 2015, 48 (1), 11 10.1186/s40659-015-0001-3. 25762051 PMC4352267

[ref39] Huang L.; Wang Q.; Zhou Q.; Ma L.; Wu Y.; Liu Q.; Wang S.; Feng Y. Cadmium uptake from soil and transport by leafy vegetables: A meta-analysis. Environ. Pollut. 2020, 264, 114677 10.1016/j.envpol.2020.114677. 32388299

[ref40] Riaz M.; Kamran M.; Rizwan M.; Ali S.; Parveen A.; Malik Z.; Wang X. Cadmium uptake and translocation: Selenium and silicon roles in Cd detoxification for the production of low Cd crops: A critical review. Chemosphere 2021, 273, 129690 10.1016/j.chemosphere.2021.129690. 33524757

[ref41] Bhat J. A.; Shivaraj S. M.; Singh P.; Navadagi D. B.; Tripathi D. K.; Dash P. K.; Solanke A. U.; Sonah H.; Deshmukh R. Role of Silicon in Mitigation of Heavy Metal Stresses in Crop Plants. Plants 2019, 8 (3), 71 10.3390/plants8030071. 30901942 PMC6473438

[ref42] Zaman Q. U.; Rashid M.; Nawaz R.; Hussain A.; Ashraf K.; Latif M.; Heile A. O.; Mehmood F.; Salahuddin S.; Chen Y. Silicon Fertilization: A Step towards Cadmium-Free Fragrant Rice. Plants 2021, 10 (11), 2440 10.3390/plants10112440. 34834803 PMC8623705

[ref43] Lesharadevi K.; Parthasarathi T.; Muneer S. Silicon biology in crops under abiotic stress: A paradigm shift and cross-talk between genomics and proteomics. J. Biotechnol. 2021, 333, 21–38. 10.1016/j.jbiotec.2021.04.008. 33933485

[ref44] Basu S.; Kumar G. Exploring the significant contribution of silicon in regulation of cellular redox homeostasis for conferring stress tolerance in plants. Plant Physiol. Biochem. 2021, 166, 393–404. 10.1016/j.plaphy.2021.06.005. 34153883

[ref45] Pavlovic J.; Kostic L.; Bosnic P.; Kirkby E. A.; Nikolic M. Interactions of Silicon With Essential and Beneficial Elements in Plants. Front. Plant Sci. 2021, 12, 697592 10.3389/fpls.2021.697592. 34249069 PMC8261142

[ref46] Lide D. R.; Frederikse H. P. R.. CRC Handbook of Chemistry and Physics, 75th ed.; CRC Press: Boca Raton, 1995; pp 8 44.

[ref47] Khan I.; Awan S. A.; Rizwan M.; Ali S.; Hassan M. J.; Brestic M.; Zhang X.; Huang L. Effects of silicon on heavy metal uptake at the soil-plant interphase: A review. Ecotoxicol. Environ. Saf. 2021, 222, 112510 10.1016/j.ecoenv.2021.112510. 34273846

[ref48] Sarai N. S.; Levin B. J.; Roberts J. M.; Katsoulis D. E.; Arnold F. H. Biocatalytic Transformations of Silicon—the Other Group 14 Element. ACS Cent. Sci. 2021, 7, 944–953. 10.1021/acscentsci.1c00182. 34235255 PMC8227617

[ref49] Puppe D.; Kaczorek D.; Stein M.; Schaller J. Silicon in Plants: Alleviation of Metal(loid) Toxicity and Consequential Perspectives for Phytoremediation. Plants 2023, 12 (13), 2407 10.3390/plants12132407. 37446968 PMC10346223

[ref50] Głowacka K.; Źróbek-Sokolnik A.; Okorski A.; Najdzion J. The effect of cadmium on the activity of stress-related enzymes and the ultrastructure of pea roots. Plants 2019, 8, 413 10.3390/plants8100413. 31615032 PMC6843902

[ref51] Cruzado Tafur E.; Orzoł A.; Gołębiowski A.; Pomastowski P.; Cichorek M.; Olszewski J.; Walczak-Skierska J.; Buszewski B.; Szultka-Młyńska M.; Głowacka K. Metal tolerance and Cd phytoremoval ability in Pisum sativum grown in spiked nutrient solution. J. Plant Res. 2023, 136, 931–945. 10.1007/s10265-023-01493-1. 37676608 PMC10587304

[ref52] Hoagland D. R.; Arnon D. I.. The Water-Culture Method for Growing Plants without Soil. In Circular 347; California Agricultural Experiment Station, 1950.

[ref53] Rahman M. F.; Ghosal A.; Alam M. F.; Kabir A. H. Remediation of cadmium toxicity in field peas (*Pisum sativum* L.) through exogenous silicon. Ecotoxicol. Environ. Saf. 2017, 135, 165–172. 10.1016/j.ecoenv.2016.09.019. 27736676

[ref54] Fimbres-Acedo Y. E.; Traversari S.; Cacini S.; Costamagna G.; Ginepro M.; Massa D. Testing the Effect of High pH and Low Nutrient Concentration on Four Leafy Vegetables in Hydroponics. Agronomy 2023, 13 (1), 41 10.3390/agronomy13010041.

[ref55] Velazquez-Gonzalez R. S.; Garcia-Garcia A. L.; Ventura-Zapata E.; Barceinas-Sanchez J. D. O.; Sosa-Savedra J. C. A Review on Hydroponics and the Technologies Associated for Medium- and Small-Scale Operations. Agriculture 2022, 12 (5), 646 10.3390/agriculture12050646.

[ref56] Salam U.; Ullah S.; Tang Z. H.; Elateeq A. A.; Khan Y.; Khan J.; Khan A.; Ali S. Plant Metabolomics: An Overview of the Role of Primary and Secondary Metabolites against Different Environmental Stress Factors. Life 2023, 13 (3), 706 10.3390/life13030706. 36983860 PMC10051737

[ref57] Swain R.; Sahoo S.; Behera M.; Rout Gyana R. Instigating prevalent abiotic stress resilience in crop by exogenous application of phytohormones and nutrient. Front. Plant Sci. 2023, 14, 1104874 10.3389/fpls.2023.1104874. 36844040 PMC9947512

[ref58] Piasecka A.; Sawikowska A.; Jedrzejczak-Rey N.; Piślewska-Bednarek M.; Bednarek P. Targeted and Untargeted Metabolomic Analyses Reveal Organ Specificity of Specialized Metabolites in the Model Grass Brachypodium distachyon. Molecules 2022, 27 (18), 5956 10.3390/molecules27185956. 36144695 PMC9506550

[ref59] Wysokinski A.; Kuziemska B.; Lozak I. Heavy Metal Allocation to Pea Plant Organs (*Pisum sativum* L.) from Soil during Different Development Stages and Years. Agronomy 2023, 13 (3), 673 10.3390/agronomy13030673.

[ref60] Yang X.; Lu M.; Wang Y.; Wang Y.; Liu Z.; Chen S. Response Mechanism of Plants to Drought Stress. Horticulturae 2021, 7 (3), 50 10.3390/horticulturae7030050.

[ref61] Zhao Y.; Wang J.; Huang W.; Zhang D.; Wu J.; Li B.; Li M.; Liu L.; Yan M. Abscisic-Acid-Regulated Responses to Alleviate Cadmium Toxicity in Plants. Plants 2023, 12 (5), 1023 10.3390/plants12051023. 36903884 PMC10005406

[ref62] Mininni A. N.; Tuzio A. C.; Brugnoli E.; Dichio B.; Sofo A. Carbon isotope discrimination and water use efficiency in interspecific Prunus hybrids subjected to drought stress. Plant Physiol. Biochem. 2022, 175, 33–43. 10.1016/j.plaphy.2022.01.030. 35176579

[ref63] Guo Z.; Gao Y.; Yuan X.; Yuan M.; Huang L.; Wang S.; Liu C.; Duan C. Effects of Heavy Metals on Stomata in Plants: A Review. Int. J. Mol. Sci. 2023, 24 (11), 9302 10.3390/ijms24119302. 37298252 PMC10252879

[ref64] Zhou H.; Zhu W.; Yang W.-T.; Gu J.-F.; Gao Z.-X.; Chen L.-W.; Du W.-Q.; Zhang P.; Peng P.-Q.; Liao B.-H. Cadmium uptake, accumulation, and remobilization in iron plaque and rice tissues at different growth stages, Ecotoxicol. Environ. Saf. 2018, 152, 91–97. 10.1016/j.ecoenv.2018.01.031. 29407786

[ref65] Leita L.; De Nobili M.; Cesco S.; Mondini C. Analysis of intercellular cadmium forms in roots and leaves of bush bean. J. Plant Nutr. 1996, 19, 527–533. 10.1080/01904169609365140.

[ref66] Cuypers A.; Vanbuel I.; Iven V.; Kunnen K.; Vandionant S.; Huybrechts M.; Hendrix S. Cadmium-induced oxidative stress responses and acclimation in plants require fine-tuning of redox biology at subcellular level. Free Radical Biol. Med. 2023, 199, 81–96. 10.1016/j.freeradbiomed.2023.02.010. 36775109

[ref67] Ahmed S. R.; Anwar Z.; Shahbaz U.; Skalicky M.; Ijaz A.; Tariq M. S.; Zulfiqar U.; Brestic M.; Alabdallah N. M.; Alsubeie M. S.; et al. Potential Role of Silicon in Plants Against Biotic and Abiotic Stresses. Silicon 2023, 15, 3283–3303. 10.1007/s12633-022-02254-w.

[ref68] Khan I.; Awan S. A.; Rizwan M.; Brestic M.; Xie W. Silicon: An essential element for plant nutrition and phytohormones signaling mechanism under stressful conditions. Plant Growth Regul. 2023, 100, 301–319. 10.1007/s10725-022-00872-3.

[ref69] Rastogi A.; Yadav S.; Hussain S.; Kataria S.; Hajihashemi S.; Kumari P.; Yang X.; Brestic M. Does silicon really matter for the photosynthetic machinery in plants...?. Plant Physiol. Biochem. 2021, 169, 40–48. 10.1016/j.plaphy.2021.11.004. 34749270

[ref70] Awan S. A.; Ilyas N.; Khan I.; Raza M. A.; Rehman A. U.; Rizwan M.; Rastogi A.; Tariq R.; Brestic M. Bacillus siamensis Reduces Cadmium Accumulation and Improves Growth and Antioxidant Defense System in Two Wheat (*Triticum aestivum* L.) Varieties. Plants 2020, 9, 878 10.3390/plants9070878. 32664464 PMC7411916

[ref71] Rizwan M.; Ali S.; Ali B.; Adrees M.; Arshad M.; Hussain A.; Zia U. R. M.; Waris A. A. Zinc and iron oxide nanoparticles improved the plant growth and reduced the oxidative stress and cadmium concentration in wheat. Chemosphere 2019, 214, 269–277. 10.1016/j.chemosphere.2018.09.120. 30265934

[ref72] Zheng Y.; Wang X.; Cui X.; Wang K.; Wang Y.; He Y. Phytohormones regulate the abiotic stress: An overview of physiological, biochemical, and molecular responses in horticultural crops. Front. Plant Sci. 2023, 13, 1095363 10.3389/fpls.2022.1095363. 36684767 PMC9853409

[ref73] Ranjan A.; Rajput V. D.; Minkina T.; Bauer T.; Chauhan A.; Jindal T. Nanoparticles induced stress and toxicity in plants. Environ. Nanotechnol. Monit. Manag. 2021, 15, 100457 10.1016/j.enmm.2021.100457.

[ref74] Lama-Muñoz A.; Contreras M. D. M. Extraction Systems and Analytical Techniques for Food Phenolic Compounds: A Review. Foods 2022, 11 (22), 3671 10.3390/foods11223671. 36429261 PMC9689915

[ref75] Alara O. R.; Abdurahman N. H.; Ukaegbu C. I. Extraction of phenolic compounds: A review. Curr. Res. Food Sci. 2021, 4, 200–214. 10.1016/j.crfs.2021.03.011. 33899007 PMC8058613

[ref76] Cowan M. M. Plant products as antimicrobial agents. Clin. Microbiol. Rev. 1999, 12 (4), 564–582. 10.1128/CMR.12.4.564. 10515903 PMC88925

[ref77] Nascimento G. G. F.; Locatelli J.; Freitas P. C.; Silva G. L. Antibacterial activity of plant extracts and phytochemicals on antibiotic-resistant bacteria. Braz. J. Microbiol. 2000, 31 (4), 247–256. 10.1590/S1517-83822000000400003.

[ref78] Hadrich F.; Arbi M.; Boukhris M.; Sayadi S.; Cherif S. Valorization of the Peel of Pea: *Pisum sativum* by Evaluation of Its Antioxidant and Antimicrobial Activities. J. Oleo Sci. 2014, 63 (11), 1177–1183. 10.5650/jos.ess14107. 25354878

