# Supplementary material for: Silicon’s Influence on Polyphenol and Flavonoid Profiles in Pea (Pisum sativum L.) under Cadmium Exposure in Hydroponics: A Study of Metabolomics, Extraction Efficacy, and Antimicrobial Properties of Extracts
Source: ACS Omega. 2024 Mar 18;9(13):14899–910. doi: 10.1021/acsomega.3c08327 (PMC10993280; doi:10.1021/acsomega.3c08327)
Supplement: Supplementary file 1 — ao3c08327_si_001.pdf [file ao3c08327_si_001.pdf]

Silicon's Influence on Polyphenol and Flavonoid Profiles in Pea (Pisum sativum L.) Under Cadmium Exposure in Hydroponics: A Study of Metabolomics, Extraction Efficacy, and Antimicrobial Properties of Extracts

Justyna Walczak-Skierska[1*], Aneta Krakowska-Sieprawska[2], Fernanda Monedeiro[1], Michał Złoch[1], Paweł Pomastowski[1], Mateusz Cichorek[2], Jacek Olszewski[3], Katarzyna Głowacka[2], Gaja Gużewska[4], Małgorzata Szultka-Młyńska[4]

[1]Centre for Modern Interdisciplinary Technologies, Nicolaus Copernicus University, Wilenska 4, 87-100 Torun, Poland

[2]Department of Plant Physiology, Genetics and Biotechnology, University of Warmia and Mazury in Olsztyn, Oczapowskiego 1a, 10-719 Olsztyn, Poland

[3]Experimental Education Unit, University of Warmia and Mazury in Olsztyn, Plac Łódzki 1, 10-721 Olsztyn, Poland

[4]Department of Environmental Chemistry and Bioanalytics, Faculty of Chemistry, Nicolaus Copernicus University, Gagarin 7, 87-100 Torun, Poland

Correspondence: walczak-skierska@umk.pl

Table S1. MRM analysis of 15 investigated compounds in the HPLC-QqQ MS analysis

Compounds name	MRM	Q1 [V]	CE [eV]	Q3 [V]	Polarity
Flavon	223.00 → 121.50	-17	-35	-13	+
Hesperidin	303.00 → 153.15	-20	-26	-21	+
Chlorogenic acid	353.15 → 191.20	8	35	16	-
Gallic acid	169.05 → 79.00	9	35	14	-
Caffeic acid	179.10 → 134.10	14	35	6	-
Ferulic acid	193.10 → 133.10	13	35	14	-
Sinapic acid	223.05 → 121.05	14	35	9	-
Catechin	289.10 → 123.10	14	35	8	-
Biochanin a	283.10 → 211.50	9	35	18	-
Esculin	339.10 → 177.20	16	35	12	-
Quercetin	301.10 → 227.10	19	35	13	-
Rutin	609.00 → 300.00	15	35	17	-
Luteolin	285.10 → 133.10	16	35	16	-
Apigenin	269.15 → 117.05	16	35	15	-
Salicylic acid	137.00 → 93.00	14	35	8	-

Table S2. Regression equation, LOD and LOQ of 15 investigated compounds

Compounds	Linear regression data			LOD [µg/mL]	LOQ [µg/mL]
	Regression equation	r	Range [µg/mL]		
Flavon	y = 9287460x + 173107	0.9994	2.5-0.00005	0.000015	0.000049
Hesperidin	y = 18561260x + 402624	0.9999	2.5-0.0001	0.000025	0.0000082
Chlorogenic acid	y = 152046x + 1458	0.9995	2.5-0.005	0.001	0.0033
Gallic acid	y = 23562x + 531	0.9994	2.5-0.01	0.001	0.0033
Caffeic acid	y = 262776x + 6932	0.9984	2.5-0.001	0.0005	0.00165
Ferulic acid	y = 24980x + 956	0.9991	2.5-0.05	0.005	0.0165
Sinapic acid	y = 123016x + 2315	0.9991	2.5-0.005	0.001	0.0033
Catechin	y = 201705x + 1298	0.9999	2.5-0.005	0.001	0.0033
Biochanin a	y = 3052242x + 30743	0.9993	2.5-0.00005	0.000025	0.000082
Esculin	y = 4206778x + 157720	0.9971	2.5-0.0001	0.00005	0.00016
Quercetin	y = 52420x + 3194	0.9985	2.5-0.005	0.001	0.0033
Rutin	y = 3172155x + 43579	0.9995	2.5-0.00005	0.000025	0.0000082
Luteolin	y = 4973844x + 269390	0.9993	2.5-0.00005	0.000025	0.000082
Apigenin	y = 3204973x + 37850	0.9993	2.5-0.0005	0.0001	0.00033
Salicylic acid	y = 529395x + 13162	0.9992	2.5-0.005	0.0005	0.00165
